# An Integrative Review of Community-Based Mental Health Interventions Among Resettled Refugees from Muslim-Majority Countries

**DOI:** 10.1007/s10597-022-00994-y

**Published:** 2022-06-25

**Authors:** Hafifa Siddiq, Ahmad Elhaija, Kenneth Wells

**Affiliations:** 1 School of Nursing, Charles R. Drew University of Medicine and Science, 1748 E. 118th St., Los Angeles, CA 90059 USA; 2 Division of General Internal Medicine and Health Services Research, University of California, Los Angeles, 1100 Glendon Ave. Suite 900, Los Angeles, 90024 USA; 3 University of California, Los Angeles, 1100 Glendon Ave. Suite 900, Los Angeles, CA 90024 USA; 4 Jane and Terry Semel Institute for Neuroscience and Human Behavior, University of California, Los Angeles, 10920 Wilshire Blvd., Suite 300, Los Angeles, CA 90024 USA; 5 Department of Psychiatry and Biobehavioral Sciences David Geffen School of Medicine, University of California, Los Angeles, 10920 Wilshire Blvd., Suite 300, Los Angeles, CA 90024 USA; 6 Department of Health Policy and Management, Fielding School of Public Health, University of California , Los Angeles, 10920 Wilshire Blvd., Suite 300, Los Angeles, CA 90024 USA

**Keywords:** Refugees, Community-based participatory research, Mental health, Psychosocial wellbeing, Immigrant

## Abstract

Resettled refugees from Muslim-majority countries are underrepresented in research and meeting their mental health needs remains a challenge for countries of resettlement. In this integrative review, we synthesize community-based mental health interventions using an ecological framework. Eleven relevant studies were identified using PubMed and PsychInfo database. Most interventions focus on micro-system level factors like promoting integration and social connections suggest improvement of outcomes including depression, anxiety, and psychological distress. Studies suggest how mental health programs addressing psychosocial wellbeing improves outcomes across ecological levels through: (1) early screening upon resettlement; (2) education and raising awareness of mental health; and (3) engagement of refugees in local community social support systems. Largely qualitative studies suggest benefits of engagement and education program for refugees, but there is a need for high quality, rigorous mental health intervention studies with resettled refugees with explicit attention to equitable and collaborative partnerships across multiple sectors in the community.

## Introduction

There are an unprecedented 80 million people forcibly displaced globally. Refugees from Muslim-majority countries, including Afghanistan, Iraq, Somalia, and Syria, represent the largest refugee-producing countries in the world (United Nations High Commissioner Refugees, 2019). Since the passage of the Refugee Act in 1980, over three million refugees have made their way to the United States (U.S.), including thousands of Muslim refugees escaping war, violence, or political instability from predominantly the Middle East/North Africa (MENA) and South Asian regions. Political turmoil, violence, and persecution increase refugees resettling in the U.S. and other high-income countries. As forcibly displaced migrants continue to reach unprecedented numbers, meeting the mental health needs of refugees resettling in the U.S. and other resettlement countries remains a challenge.

Refugees from Muslim-majority countries comprise over half of refugees resettled to the U.S. yet are under-represented in health promotion and mental health research. The UNHCR defines a refugee as a person who has fled war, violence, conflict, or persecution and has crossed an international border to find safety in another country (United Nations High Commissioner Refugees, 2019). Refugees experience a high prevalence of mental health issues, including psychological distress, depression, anxiety, and post-traumatic stress (Blackmore et al., 2020; Bogic et al., 2015). Despite limited prevalence data, an estimated one in three refugees fleeing violence and war-torn countries experience depression, anxiety, and PTSD from pre-migration exposures to post-migration resettlement (Blackmore et al., 2020).

When refugees resettle in new contexts, they are left with the lingering trauma of forced migration, violence, and persecution compounded by ongoing stressors in the post-migration context. In addition to economic stressors, resettled refugees may experience the breakdown of family and social ties, social isolation, discrimination, and acculturative stress (Szaflarski & Bauldry, 2019). They now must adapt to a new culture, including a new social and healthcare system. Additionally, heightened Islamophobia, defined as the fear of Muslims, or anti-Muslim prejudice, has increased across resettlement countries (Kishi, 2017). Negative stereotypes against Muslims have resulted in bias and discrimination against Muslims and other ethnic groups ascribed to a Middle Eastern race or ‘Muslim identity.’

For refugees, immigrants, and first- or second-generation Muslims in the U.S., the distress associated with discrimination may correlate with adverse health outcomes (Samari et al., 2018). Hate speech, denial of employment in the workplace, and frequent negative media portrayals of Muslims may result in psychological distress and trauma (Ali et al., 2021). State-inflicted violence on Muslims, including restrictive immigration and refugee policies, can also negatively impact Muslim Americans’ willingness to seek social and health services and trust in the government (Samari et al., 2018; Samuels, 2021). Compounding these issues for many refugees or immigrants with precarious visa status are broader socio-political climate and policies, including the recent economic stress resulting from the COVID-19 pandemic, the temporary closures of borders and refugee resettlement and US Customs and Immigration Services (USCIS) offices, and family separation (Siddiq & Rosenberg, 2021). These stressors and traumatic events affecting immigrant and refugee communities may interact with existing trauma and prolong mental health issues (Alemi et al., 2020).

Refugees experience social and cultural influences on their beliefs about mental health and help-seeking behaviors and may underutilize mental health services (Satinsky et al., 2019). Barriers to mental health care for this population include limited English language proficiency, cultural beliefs about mental health, lack of access to reliable transportation, and mental health stigma (Salami et al., 2019; van der Boor & White, 2020). Refugees’ ability to access mental health services within the immediate resettlement phase in the U.S. is mainly dependent on factors like early screening and referral to services, but also can be influenced by stigma towards mental health and limited knowledge about mental health treatment (Phillips & Lauterbach, 2017). Islam may also be a significant part of cultural identity and plays an essential role in influencing and shaping attitudes on mental health and recognizing and coping with mental health. Additionally, Muslim gender norms and gender differences in attitudes towards mental health, coping mechanisms, and help-seeking behaviors also impact mental health help-seeking behaviors in this population (Al-Nuaimi & Qoronfleh, 2020; Ali et al., 2021; Assari & Lankarani, 2017; Hajak et al., 2021). Barriers to services for unmet mental health needs may severely impact refugees’ ability to cope with and function in aspects of their life, including employment, education, and building family and social connections.

The U.S. resettlement system largely overlooks the mental health resources required by refugees. Instead, the current system requires refugees to attain specific requirements to retain their temporary status. For example, refugees must find employment within a given time (usually around 90 days) and undergo a domestic medical exam. Screening for mental health and chronic health issues is not mandated the same way infectious diseases are for this population. The focus on refugees finding employment immediately upon resettlement may also compound existing stress related to acculturation or unaddressed mental health issues, especially if refugees do not speak English.

Additionally, it is essential to highlight the social stigma surrounding accessing and using mental health services in the Muslim community. For many Muslims, help-seeking with family, friends, and faith leaders for mental health-related concerns occur before obtaining care from mental health specialists (Phillips & Lauterbach, 2017). Therefore, Islamic clergy within religious community settings may be considered a first-line defense in addressing mental health issues in Muslim communities (Syed et al., 2020). Additionally, access to mental health resources for refugee and immigrant communities decreased during the (COVID-19) pandemic (Benjamen et al., 2021). Many services have become remote, requiring technology that many refugees and immigrants do not have the financial resources to obtain or know about using the technology necessary.

The increasing diversity of refugees resettling in the U.S. poses new challenges for mental health service providers in providing culturally tailored and language-specific services. A prior review of the literature examining therapeutic interventions among diverse groups of refugees suggests that resettlement interventions targeting culturally homogeneous refugee samples demonstrate moderate to large outcome effects on aspects of traumatic stress and anxiety reduction (Murray et al., 2010). Critical elements of delivering mental health services for this population include accessibility and cultural appropriateness. Partnerships with the community and multi-sector collaborations can help address diverse health and social inequities by promoting social wellbeing and addressing the social determinants of mental health for vulnerable immigrant populations (Castillo et al., 2019). However, scant literature describes interventions that utilize a culturally tailored approach to the mental health needs of refugees from Muslim-majority countries.

To address this gap in the literature, we sought to synthesize studies using an ecological framework to characterize community-based mental health interventions that aim to address the needs of this population. We examine the following:

1. Study characteristics, including the sociocultural factors and ecological level targeted for interventions.
2. How studies measure and conceptualize *mental health outcomes* and *access to mental health care*.
3. The extent of community engagement in developing and implementing interventions.

## Framework

We use Bronfenbrenner’s (1977) ecological model to examine the environmental contexts in which interventions and programs embed within. Within this model, interrelated environmental systems exist to affect individual-level outcomes and divide a person’s environment into four different systems: the microsystem, the mesosystem, the exosystem, and the macrosystem (Bronfenbrenner, 1977). The individual level is the core of the bioecological model and consists of factors like sex and age characteristics that influence health outcomes for individuals. This model also allows the identification of gaps in the implementation of prevention and early intervention efforts occurring across different levels. The microsystem is the most influential level and is the most immediate environment, including family, work, and religious and social group settings. The mesosystem refers to the interconnections between systems (for example, between family and community, mosque and community, etc.). The exosystem accounts for environmental contexts, including relevant policies and organizations that affect refugees, but not directly. And lastly, the macrosystem is the overarching cultural values and norms (for example, gender roles, social identity, and other attitudes towards migrants). Important to note that each system influences the individual’s development (or health outcome) and the power of the individual to reflect the change in each ecological context.

**Fig. 1:**
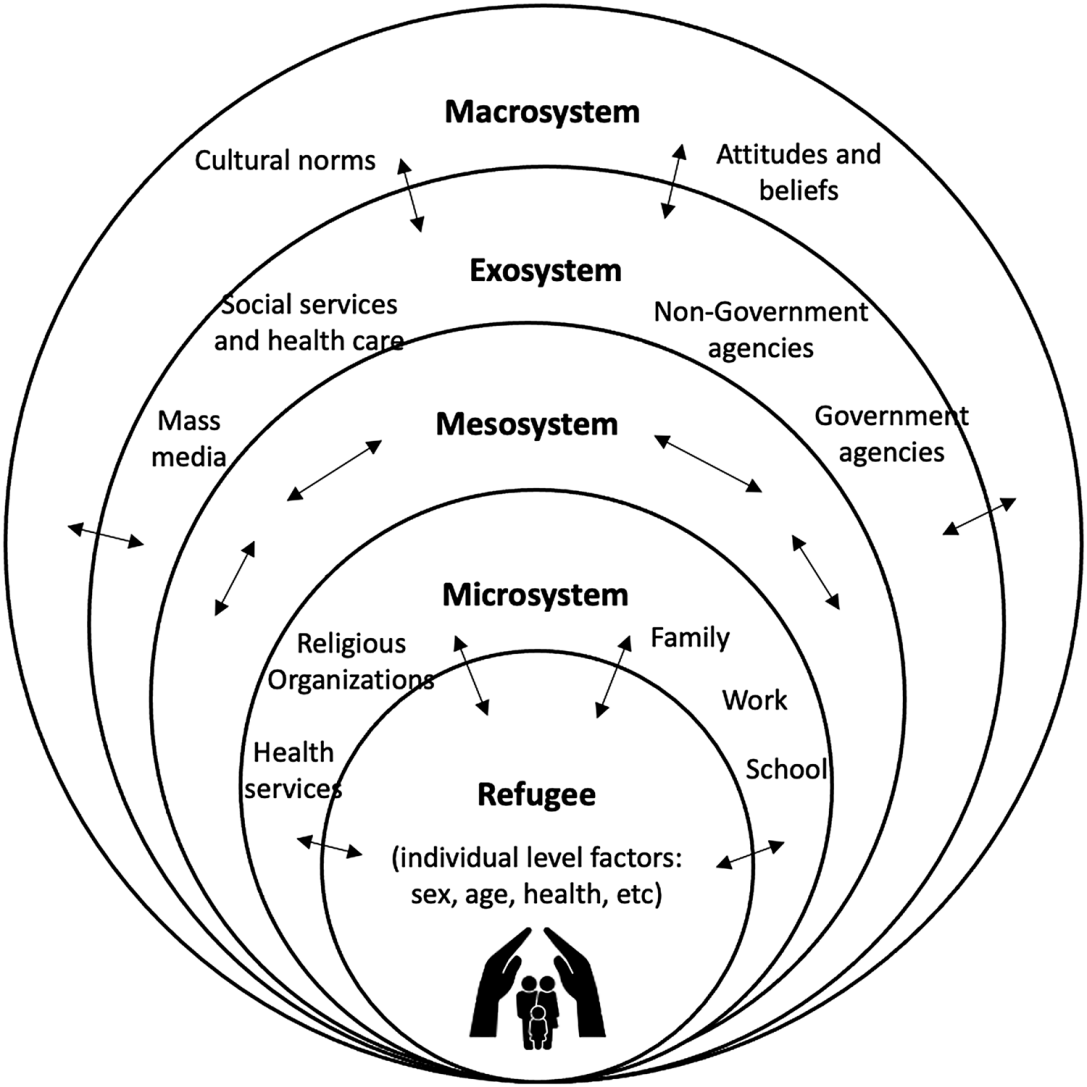
Ecological model and levels of influences. This model was adapted from Bronfenbrenner’s bioecological model (1977)

The applicability of this model beyond human development has been utilized by researchers across multiple disciplines, including how Bronfenbrenner’s ecological systems model can be a useful framework in mapping out research that represents qualitative, quantitative, and mixed-methods research (Onwuegbuzie et al., 2013). Onwuegbuzie et al. (2013) conceptualized how studies may be organized within the microsystem where persons or groups are studied within their immediate environment. They identify how studies within the mesosystem involve research with persons or groups within other systems that they spend time in. Studies within the exo-system involve research where one or more persons or groups are examined within systems that may be influenced by but do not play an active role within. They also describe studies in the macro-system that would involve research where one or more persons or groups are within the larger cultural world or society surrounding them (Onwuegbuzie et al., 2013). See Fig. 1 for an adapted representation of the model.

## Methods

This study uses an integrative review methodology, appropriate for this study, as it identifies the breadth of the topic and allows for the inclusion of interventions using diverse methods (Whittemore & Knafl, 2005). An integrative review method is an approach that allows for the inclusion of various methodologies, including experimental and non-experimental research. The entire review process followed the following phases: problem identification, literature search, data evaluation, data analysis, and presentation of findings.

### Study Selection Process

Relevant journal articles were identified through electronic sources such as PubMed and PsycINFO. We applied a string combination of search terms to include “community” AND “refugee” AND “mental health.” Potentially relevant studies were subjected to a full-text review for data extraction purposes. We included peer-reviewed journal articles that employed qualitative, quantitative, and mixed-methods approaches to examine community-based interventions addressing mental health. We sought after studies that included refugee participants from Muslim-majority countries resettled in high-income countries. Due to the limited number of studies with homogenous samples of refugee populations, and the variable use of the terms’ immigrant’ and ‘refugee’ to identify such populations, we included studies that broadly identified samples from high refugee-producing countries with predominantly Muslim populations. We also included studies that may have mixed refugee samples, as long as the samples include refugees from Muslim-majority countries. We limited our selection of articles to primarily adult refugees’ mental health interventions based in the U.S. and other developed nations while choosing journal articles published between 2009 and 2020. We decided on the starting year of 2009 as we wanted our data to be on recent community-based interventions relevant to refugee resettlement in the past 10 years.

**Fig. 2:**
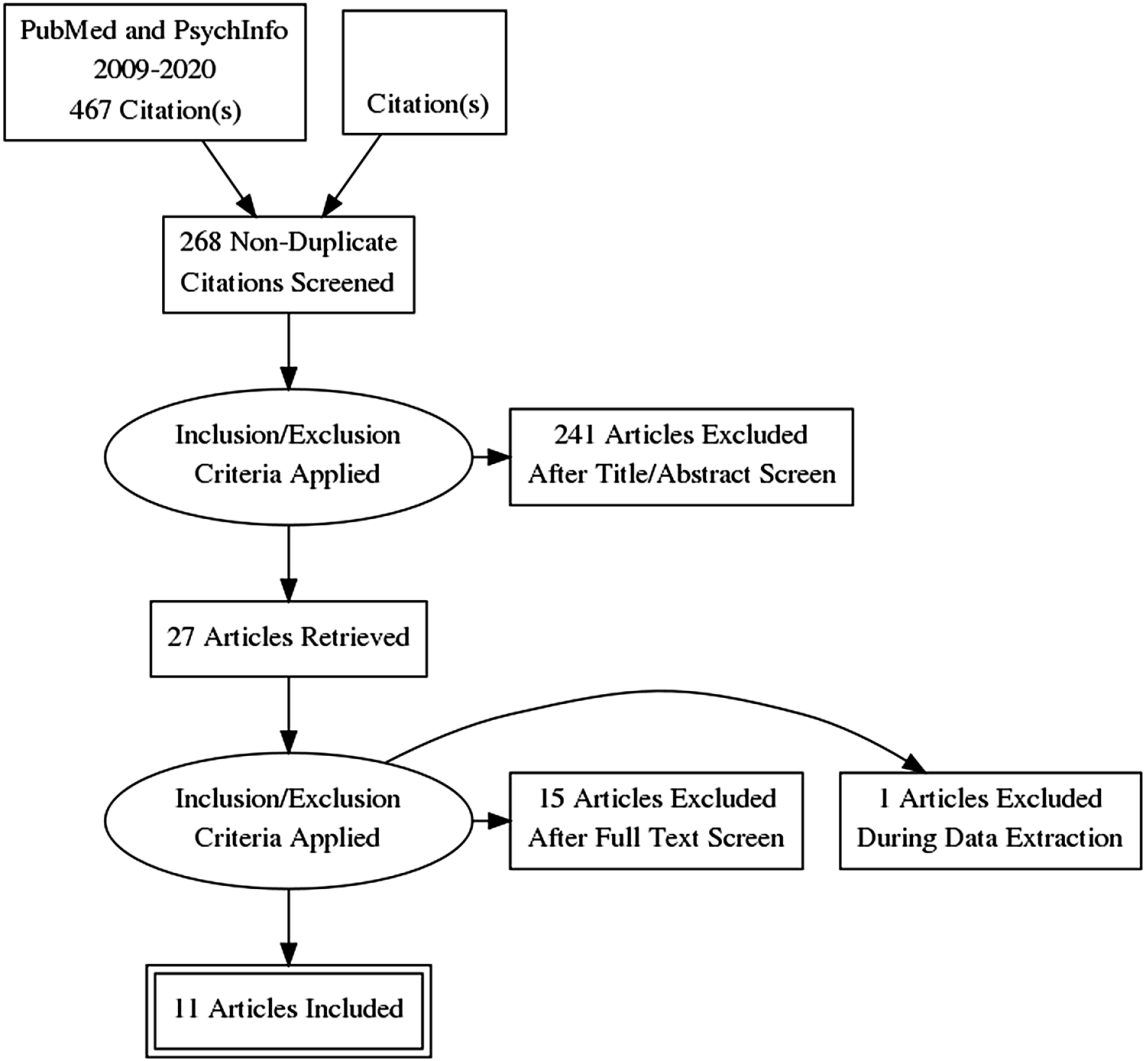
PRISMA adapted from Moher et al. (2009)

The initial literature search using two search engines yielded 268 potentially relevant articles but was not about the population of interest. After screening titles and abstracts, 27 articles met our inclusion criteria. A full-text review of 27 studies resulted in excluding 15 articles that did not meet our inclusion criteria. These studies include a case study of a community day center for asylum seekers as an early mental health intervention, the development of a professional training program for mental health clinicians treating new Iraqi refugee communities, and a review of refugee mental health interventions following resettlement. While conducting the data evaluation process, we excluded a study that included an intervention for Palestinian refugees in Lebanon due to Lebanon’s status as a middle-income nation. Another study focused on refugee youth. See Fig. 2 for the inclusion and exclusion PRISMA flow chart.

### Data Analysis

We extracted information on population characteristics, study setting, study purpose, methods of conducting the study, sample demographics, level of community involvement, and findings. Factors and major themes from each study were categorized within each ecological group and are discussed. There are no known conflicts of interest in the development of this study. Study characteristics are further outlined in Table 1.

**Table 1:** Study characteristics of community-based mental health interventions among resettled refugees from Muslim-majority countries

Author	Purpose	Design	Intervention	Participants	Community setting/involvement	Results/mental health outcomes
Baird et al. (2017)	To evaluate the acceptability and feasibility of a community-based, culturally tailored mental health intervention	Mixed-methods, pre-and post-test intervention design including interviews with participants	*Healthy Sudanese Families*: a 10-week educational and social support program	n = 12 South Sudanese refugee women between 29 and 66 years old	Partnership with a Sudanese Church; Church endorsed the project and gave approval to use Church space to implement project	The majority of the post-intervention Hopkins Symptom Checklist-25 (HSCL) scores were higher than the pre-intervention scores for both the anxiety and depression subscales at both individual and group levels. Eight of 15 items on the depression subscale increased post-intervention. Scores on six of the ten items on the anxiety subscale increased post-intervention. Three themes corroborate quantitative findings: knowledge and awareness of mental disorders, stigma, and empowerment
Betancourt et al. (2020)	To pilot a feasibility and acceptability trial of the home-visiting Family Strengthening Intervention	Randomized quantitative study (randomized between intervention or care as usual)	*Home-Visiting Family Strengthening Intervention for Refugees* (FSI-R)	A total of 40 Somali Bantu (n = 103 children, 58.40% female; n = 43 caregivers, 79.00% female and 40 Bhutanese (n = 49 children, 55.30% female; n = 62 caregivers, 54.00% female) families were randomized to receive FSI-R or CAU	Refugee community members were engaged at every phase of the pilot and worked as interventionists, research assistants (RAs), and community advisory board (CAB) members. Staff was from the two refugee communities and affiliated with community advocacy and local social service agencies	The program retention rate of 82.50% indicates high feasibility, and high reports of satisfaction (81.50%) indicate community acceptance. The FSI-R children reported reduced traumatic stress reactions, and caregivers reported fewer child depression symptoms compared with care as usual families
Chase & Rousseau, (2018)	An ethnographic case study of a community day center for asylum seekers as early-stage mental health intervention	Qualitative study with the analysis done through utilizing an ethnographic methodology, use of field notes and	Community Day Center	Interviews with n = 15 diverse English or French-speaking participants including nine asylum seekers, five accepted refugees (former asylum seekers), and one non-refugee staff membersAge of asylum seeker/refugee interviewees ranged from 26 to 55 years old	Researchers were unable to hire professional interpreters to assist with interviews due to budget constraints. While they had initially planned to work with volunteer interpreters, concerns over privacy expressed by potential participants and the sensitivity of interview content dissuaded them from engaging in this strategy	Interviews identified aspects of wellbeing and the salient threats to wellbeing related to safety and security, bonds and social networks, injustice and human rights violations, threats to roles and identity, and existential meaning. Participants elaborated pathways through which participation in the Day Center worked to remediate these threats, supporting how the Day Center shows significant promise as an innovative early-stage mental health intervention for precarious status migrants
Govindasamy et al. (2011)	An ethnographic process evaluation of a community support program with Sudanese refugee women	Qualitative analysis of a group interview and ethnographic process evaluation techniques	Sudanese women’s group: Exercise program	n = 12 Sudanese Refugee Women between 25 and 41 years old	The Penrith Women’s Health Centre (PWHC) and NSW Sport and Recreation co-funded the program, facilitated by a PWHC community worker and interpreter. Staff were involved in group activities that included formulation of research questions. A final report was disseminated to PWHCThe program was facilitated by a PWHC community worker andan interpreter,	Participants identified exercise benefits were not merely physical, and viewed the program positively due to educational components and the opportunity for respite. Transportation and childcare were perceived as critical components. Women identified ongoing resettlement stressors.
Uribe Guajardo et al. (2018)	To evaluate a face-to-face mental health literacy course for community-based workers providing initial help to Iraqi refugees	Qualitative study utilizing uncontrolled pre, post and follow-up design	Face-to-face mental health literacy course	n = 86 Adults Iraqi refugees. Average years of age=44.1	Participants were community-based workers, based in Western Sydney, assisting Iraqi refugees on their resettlement.	Improvements were reported in confidence of participants when helping an Iraqi refugee with PSTD and depression.
Hess et al. (2014)	Reducing mental health disparities through transformative learning: A social change model with refugees and students	Mixed methods study within-group longitudinal design	*Refugee Well-being Project*	n = 36 African adult refugees and n = 53 undergraduate students	Students involved in the study were from a local university, and the refugees in the study were contacted by a local refugee resettlement agency to describe the intervention and ask if they were interested in learning more.	Student participants reported in the RWP constituted a transformative learning experience, through which refugees and students came to new understandings of the relationship between social inequities and well-being
Polcher et al. (2016)	To initiate early mental health screening for newly resettled adult refugees	Non-randomized quantitative study	Pilot Project	n = 178 Adult refugees from 8 countries (Bhutan, Iraq, Somalia, Congo, Sudan, Burma, Iran, Eritrea)	Family Healthcare a community health clinic; contracted with Family Healthcare to provide initial refugee health screening and treatment within the first few months after arrival	Iraq Refugees had significantly higher scores than other refugees in screening. Only 50% of refugees who screened positive on the RHS-15 agreed for treatment for their psychological distress
Salt et al. (2017)	To pilot the Refugee Health Screener-15 (RHS-15) to assess mental health, the intervention, and to identify internal and structural barriers affecting resettlement with a refugee women’s sewing group	Non-randomized quantitative collaborative study utilizing social ecological approach.	Pathways to Wellness (PW) intervention	n = 12 refugee women from five countries (Somalia, Iran, Nepal, Burma, Chad)	Lutheran Community Services Northwest (LCSNW) in Seattle worked with collaborators, professional translators, and the refugee community to develop, test, and validate the RHS-15 as an effective screener for mental health. A community partnership was established with the Center for Refugee Services (CRS). The CRS assisted with the recruitment of interpreters and translators for the study.	80% of the participants at baseline RHS-15 scored required referrals for follow-up. There was no statistically significant difference in the total scores for the baseline RHS-15 survey (M = 25.00, SD = 16.33) and the post-intervention RHS-15 survey (M = 27.00, SD = 13.76). The baseline intervention survey (M = 33.20, SD = 7.81) and post-intervention survey (M = 31.90, SD = 6.11) difference in scores was not significant.
Shah et al. (2019)	To explore refugees’ perceptions of the impact of communication through ICTs on their mental health, the exercise of agency by refugees within the context of ICT use	The qualitative component of each interview involved a semi-structured interview guide. Constructivist grounded theory guided the qualitative analysis	Data from the *Refugee Well-being Project*	n = 290 Adult Refugees from Afghanistan, Iraq, Syria and the Great Lakes region of Africa (Burundi, Democratic Republic of Congo and Rwanda)	Unclear	Participants described a range of mental health effects. ICTs, as channels of communication between separated families, were a major source of emotional social support and mental well-being for a large number of refugee participants. However, for some participants, the communication process with separated family members through digital technology was mentally and emotionally difficult.
Slewa-Younan et al. (2020)	An evaluation of a mental health literacy course for Arabic speaking religious and community leaders in Australia: effects on posttraumatic stress disorder related knowledge, attitudes and help-seeking	Uncontrolled, pre-post intervention design utilizing a survey questionnaire.	Mental health intervention: Key aspects of mental health literacy, help-seeking intentions and levels of general psychological distress were assessed, by means of a self-report survey, pre-intervention, (immediately) post-intervention and 3 months following intervention	n = 33 Arabic-speaking refugees	Intervention was delivered in Arabic by experienced bilingual health educators and/or mental health clinicians. The program content, which was developed by the authors in partnership with the NSW Refugee Health Service, was designed to be culturally sensitive and to be interactive with group discussion encouraged	Improvements in most aspects of mental health literacy assessed were found immediately post-intervention and at follow-up, although only changes relating to stigmatizing attitudes were statistically significant. Additionally, a statistically significant decrease in participants’ levels of general psychological distress was observed immediately following the intervention, and this decrease was sustained at 3 months follow-up
Walker et al. (2015)	Social Connectedness and mobile phone use among refugee women in Australia	Mixed methods design utilizing questionnaires and interviews	Pilot Project	n = 111 refugee women 19 years and older from 3 countries (Sudan, Afghanistan, Burma)	Potential participants were invited by Afghan, Burmese, and Sudanese community leaders to an information session, where the study was explained and invitations to participate was extended	Free-call phones and peer support training enables personal relationship to be formed, to deepen and provide emotional, informational and practical assistance that was of great value to them and enhanced their quality of life

### Findings

This study includes 11 community-based mental health programs that fit our inclusion criteria. These studies address screening, prevention, and access to mental health services across four ecological levels of influence. Most studies were considered micro-level research. Topics included: early screening upon resettlement, education and raising awareness of mental health, and engagement of refugees across settings (including within their homes and faith-based institutions). In contrast, no studies identified in this review represent macro-level research. Mental health was generally operationalized as outcomes related to psychosocial stressors and wellbeing. For example, outcomes of interest included assessing social integration, social connectedness, and measures for depression and depressive symptoms, anxiety, and psychological distress.

Only a few studies explicitly reported using the CBPR framework that guided the development of these interventions. The studies included in this review used non-randomized quantitative studies (n = 5), qualitative studies (n = 3), and mixed methods (n = 3) to evaluate pilot interventions. Analytic generalizations from these findings used mental health outcome measures and perceptions of refugees within their immediate environments. Largely qualitative studies suggest the benefits of engagement and education programs to improve mental health outcomes for refugees in the community setting. Most interventions (n = 7) targeted heterogeneous samples of resettled refugees from different countries of origin, including Somalia, Iraq, Iran, and Afghanistan. However, refugee participants within these study samples also included those from non-Muslim countries, including Burma, South Sudan, and others. While most studies had diverse samples of refugees, only a few (n = 4) focused on a single ethnic group (Baird et al., 2017; Betancourt et al., 2020; Hashimoto-Govindasamy & Rose, 2011; Uribe Guajardo et al., 2018). Nearly all studies were conducted within the U.S.: in Texas, Midwestern U.S., North Dakota, and New Mexico.

At the micro-system level, studies promoted social integration and addressed refugees’ social connections and interpersonal relationships with families and peers in the community to improve mental health outcomes. For example, one intervention implemented a home visiting program for refugees (Betancourt et al., 2020). This intervention promoted refugees’ interaction with caregivers through home visits and aimed to improve mental health, and promoted family relationships. In another intervention, refugees were provided with free-call phones and peer support training to promote social connectedness with existing social networks and form new social support with peers (Walker et al., 2015). Another intervention used mobile phones, the internet, and social media, to promote mental health (Shah et al., 2019). This study focuses on bridging the physical distance separating refugees and their families to decrease distress and improve emotional wellbeing. Results from this study show positive effects of digital technology to keep in touch with separated family members. These positive effects were identified as healing, encouragement, social support, and bridging the physical distance separating refugees and their families. Participants reported stress-related effects of using digital technology to keep in touch with separated families. Stressors included heightened concerns about the family’s safety and feelings of helplessness of not being able to help their distant family members (Shah et al., 2019).

Programs at the meso-level addressed refugees’ mental health outcomes through the promotion of mental health literacy among service-providers, early mental health screening in community settings, and building social connections and social integration through healthcare or informal community settings (Polcher & Calloway, 2016; Salt et al., 2017; Uribe Guajardo et al., 2018). Through partnerships between academic health systems and communities, interventions engaged community members, health care workers, peer counselors, university students, and church or religious leaders. In other interventions, refugees were encouraged to interact with other refugees through social groups and peer support training. Social groups included activities like sewing and exercise groups that were gender exclusive and were viewed positively by participants (Hashimoto-Govindasamy & Rose, 2011; Salt et al., 2017). Another study implemented a culturally-tailored intervention called Healthy Sudanese Families in a church setting that provided mental health education to address the stigma associated with mental health and help-seeking (Baird, 2017). In the Refugee Wellbeing Project, African refugees interacted with undergraduate students in a program to reduce mental health disparities (Hess et al., 2014). Two studies focused on initiating early mental health screening for this population using the Refugee Health Screener-15 (RHS-15) with a referral to treatment (Polcher & Calloway, 2016; Salt et al., 2017). In one study, only 50% of refugees who screened positive on the RHS-15 agreed to get treatment for psychological distress (Polcher & Calloway, 2016).

We identified one study within the exo-system that involves research where one or more persons or groups are examined within systems that may be influenced by but do not play an active role within. For example, community-trained workers in a Mental Health Literacy Course provide culturally sensitive help to Iraqi refugees with depression and post-traumatic stress disorder (PTSD) (Uribe Guajardo et al., 2018). This study found that the training increased community-based workers’ perceived helpfulness and confidence when helping Iraqi refugees with PTSD. While studies within the macro-system level would involve research where one or more persons or groups are within the larger cultural world or society surrounding them, we could not identify studies at the broader contextual level.

Levels of community involvement in developing these interventions also vary across studies. There are different ways resettled refugees were involved in developing the program or intervention. Among the programs for refugees from Muslim-majority countries we examined, two programs explicitly reported using a Community-Based Participatory Research (CBPR) approach to developing their pilot study. In the Home Visiting Family Intervention Program, refugee community members were engaged across phases of the pilot and worked as interventionists, research assistants (RAs), and community advisory board (CAB) members (Betancourt et al., 2020). Staff was from the two refugee communities and affiliated with community advocacy and social service agencies in the area. For example, RAs from the two communities and trained by the management staff conducted outreach via phone calls, home visits, and events organized by the program manager and community leaders. RAs recruited all participants and conducted blinded child and caregiver assessments via in-person interviews in three languages. Most studies reported language-specific interventions, interpreters, and translated materials in implementing programs. In the Pathway to Wellness intervention, community service workers from the Lutheran Community Services Northwest (LCSNW) served as consultants (Salt et al., 2017). They provided guidelines as well as scripts to assist the research team.

## Discussion

Overall, the findings of this integrative review suggest that there is a paucity of high-quality intervention studies that specifically focus on refugees from Muslim-majority countries. Programs address psychosocial factors underlying mental health that emphasizes social connectedness and assist refugees in assimilating into their new country. Through multi-sector partnerships, researchers utilized peers, community-based workers, technology, and even students at a local university, to address mental health needs. We find that community-based mental health interventions generally benefit refugees, specifically from Muslim-majority countries, in line with prior research across refugee groups (Riza et al., 2020; Williams & Thompson 2011). Similarly, a recent review of community-based healthcare interventions for refugees across a broad range of health-related issues and diverse refugee populations highlights the need to address linguistic barriers, cultural differences, close collaboration, and partnerships with stakeholders and refugee communities (Riza et al., 2020).

This study identifies interventions that highlight broader psychosocial outcomes within more immediate environmental levels (at the micro-and meso-level system) and focuses on the integration challenges experienced by newly resettled refugees. There is less attention to addressing mental health needs beyond the initial resettlement period and within the broader macro-level system. Muslim culture, religion, and mental health beliefs were generally discussed at the micro-level through education and de-stigmatization efforts. Although one intervention was implemented in a church setting, refugees’ religious affiliations were not reported. These findings suggest that developing culturally-tailored interventions targeting Muslim refugee groups within mosque settings is a critical gap in research—given that about half of the resettled refugees in the U.S. identify as Muslim.

Mental health stigma remains a significant barrier to accessing mental health care among Muslim immigrants and is an essential aspect of developing culturally-tailored community mental health interventions (Amri, 2013). Promoting social-based therapies and mental health literacy in familiar settings like within the home, mosques, and even through partnerships between healthcare settings and resettlement agency sites may help address stigma towards mental health treatment and improve the use of and access to community-based mental health services. For example, specialized interventions such as the home-visiting Family Strengthening Intervention have improved overall mental wellness to a greater degree than standard mental health care practices such as traditional therapy (Betancourt et al., 2020).

Moreover, previous research has long emphasized good communication and the need to address linguistic barriers as critical elements of interventions with resettled refugees (Riza et al., 2020). There is increasing diversity of refugees resettled within the U.S. Refugees from Muslim-majority countries make up a significant number of those resettled within the U.S. and speak a diverse range of languages, including Arabic, Farsi, Dari, and other dialects. Delivery of mental health and social services in these languages or the use of interpreter services is crucial to addressing the needs of this population. There is also a need to understand better how stigma or mental health is also captured within these languages. For example, one study reported surprising results showing post-intervention anxiety and depression scores higher than the pre-intervention scores (Baird et al., 2017). In this study, researchers indicate that increased scores may have been related to increased sensitivity, understanding, and awareness of symptoms of anxiety and depression after the completion of the intervention (Baird et al., 2017).

Community-based participatory research (CBPR) approaches have been identified as a methodology that has the potential to improve health and wellbeing in marginalized and under-resourced communities (Williams & Thompson, 2011). However, the extent to which studies reported community engagement in developing, implementing, and evaluating community-based interventions was limited. In one study, mental health-related topics for each session of the Healthy Sudanese Families program were selected through meetings with community members (Baird et al., 2017). In another study, program content was developed with the refugee-serving partner organization with the intent to design the intervention as culturally sensitive (Slewa-Younan et al., 2020). Endorsement of church and religious leaders and the involvement of refugee community members as interpreters and program facilitators increase the acceptability and relevance of these programs. While data analysis appeared to have been conducted by the research team across all studies, data triangulation through interviews was used to help explain quantitative data (Hess et al., 2014). Only a few pilot studies reported the explicit use of the CBPR approach in developing community-based interventions. As the needs of refugee communities are so diverse, efforts should be made to include refugees as partners in all stages of the research process. In a scoping review of refugee participation in the CBPR, researchers reported refugees having less involvement in data analysis, knowledge translation/dissemination, the inception of the research study, obtaining funding, or contributing to scale up initiatives (Filler et al., 2021). Building strong partnerships should also include working with refugees from diverse backgrounds and community members at different stages of the post-resettlement phase (from newly resettled to former refugees). Researchers need to collaborate with refugee communities from the inception, dissemination, and sustainability of research studies and program development.

Refugees from Muslim-majority countries represent an overlooked population in public mental health promotion, and we identify research gaps. There were limited randomized controlled trials that addressed the mental health needs of refugees from Muslim-majority countries. There is an urgent need to address the role of Islamophobia and perceived discrimination affecting this population. Muslim groups in the U.S. represent a religious minority vulnerable to discrimination that may exacerbate pre- and post-migration-related stressors (Alemi & Stempel, 2018). This has significant implications for second-generation refugees and immigrants that identify as Muslim in the U.S. A recent study examining suicide attempts of Muslims compared to other religious groups in the US found that Muslims were 2.18 (95% CI 1.13–4.20; *P* < .05) times more likely to report a lifetime suicide attempt compared with Protestants (Awaad et al., 2021). This supports prior calls by leading migrant experts worldwide to address discrimination at the health system level as a priority in policy development (Pottie et al., 2017). Additionally, more focus is needed to identify the challenges of interagency collaboration in refugee agencies–community partnerships that facilitate the use of community-based mental health services. As these partnerships evolve, there is a need to identify how these challenges can impact local and national refugee programs and policy development and evaluation.

## Limitations

Although this study includes quantitative and qualitative research, combining the literature provides a more comprehensive understanding of the phenomenon and, thus, has the potential to inform future research and policy initiatives. A limitation in this study is in comparing studies because different measurements were used for the outcomes of interventions. It is also challenging to compare research findings on heterogeneous groups of refugees from other countries. For this reason, it may be beneficial to consider classifying refugees by region of origin rather than only country of origin.

## Conclusions

The ecological model is a useful framework for developing interventions for refugees because it highlights environmental levels of influence as targets for intervention development. Overall, these studies suggest that community-based mental health care using multi-sector collaborations effectively addresses psychosocial wellbeing among resettled refugees from Muslim-majority countries. Mental health may be improved by early screening of refugees upon arrival, providing culturally- and language-tailored education, raising awareness about mental health and services, and engaging refugees in intervention development. These elements promote engagement in developing more culturally tailored mental health programs. As resettled refugees in the U.S. become more diverse, there is a critical need for high-income countries to define, evaluate, and adopt culturally appropriate and effective interventions to address mental health in this population. Therefore, including refugee perspectives in research and partnership in intervention development and evaluation will be critical to promoting refugee mental health and wellbeing. This requires increased awareness, training, and funding, to implement longitudinal interventions that work collaboratively with clients from refugee backgrounds through the stages of resettlement.

## References

[CR1] Alemi Q, Stempel C (2018). Discrimination and distress among Afghan refugees in northern California: The moderating role of pre- and post-migration factors. PLoS One.

[CR2] Alemi Q, Stempel C, Siddiq H, Kim E (2020). Refugees and COVID-19: Achieving a comprehensive public health response. Bulletin of the World Health Organization.

[CR3] Ali S, Elsayed D, Elahi S, Zia B, Awaad R (2021). Predicting rejection attitudes toward utilizing formal mental health services in Muslim women in the US: Results from the Muslims’ perceptions and attitudes to mental health study. The International Journal of Social Psychiatry.

[CR4] Al-Nuaimi SK, Qoronfleh MW (2020). Mental health and psycho-social-spiritual support for Muslim populations in emergency settings. Journal of Muslim Mental Health.

[CR5] Amri SBF (2013). Mental health help-seeking behaviors of muslim immigrants in the United States: Overcoming social stigma and cultural mistrust. Journal of Muslim Mental Health.

[CR6] Assari S, Lankarani MM (2017). Discrimination and psychological distress: Gender differences among Arab Americans. Frontiers in Psychiatry.

[CR7] Awaad R, El-Gabalawy O, Jackson-Shaheed E, Zia B, Keshavarzi H, Mogahed D, Altalib H (2021). Suicide attempts of Muslims compared with other religious groups in the US. JAMA Psychiatry.

[CR8] Baird M, Bimali M, Cott A, Brimacombe M, Ruhland-Petty T, Daley C (2017). Methodological challenges in conducting research with refugee women. Issues in Mental Health Nursing.

[CR9] Benjamen J, Girard V, Jamani S, Magwood O, Holland T, Sharfuddin N, Pottie K (2021). Access to refugee and migrant mental health care services during the first six months of the COVID-19 pandemic: A Canadian refugee clinician survey. International Journal of Environmental Research and Public Health.

[CR10] Betancourt TS, Berent JM, Freeman J, Frounfelker RL, Brennan RT, Abdi S, Maalim A, Abdi A, Mishra T, Gautam B, Creswell JW, Beardslee WR (2020). Family-based mental health promotion for somali bantu and bhutanese refugees: Feasibility and acceptability trial. The Journal of Adolescent Health.

[CR11] Blackmore R, Boyle JA, Fazel M, Ranasinha S, Gray KM, Fitzgerald G, Misso M, Gibson-Helm M (2020). The prevalence of mental illness in refugees and asylum seekers: A systematic review and meta-analysis. PLoS Medicine.

[CR12] Bogic M, Njoku A, Priebe S (2015). Long-term mental health of war-refugees: A systematic literature review. BMC International Health and Human Rights.

[CR13] Bronfenbrenner U (1977). Toward an experimental ecology of human development. American Psychologist.

[CR14] Castillo EG, Ijadi-Maghsoodi R, Shadravan S, Moore E, Mensah MO, Docherty M, Aguilera Nunez MG, Barcelo N, Goodsmith N, Halpin LE, Morton I, Mango J, Montero AE, Rahmanian Koushkaki S, Bromley E, Chung B, Jones F, Gabrielian S (2019). Community interventions to promote mental health and social equity. Current Psychiatry Reports.

[CR15] Chase Liana E., Rousseau Cécile (2018). Ethnographic case study of a community day center for asylum seekers as early stage mental health intervention.. American Journal of Orthopsychiatry.

[CR16] Filler Tali, Benipal Pardeep Kaur, Torabi Nazi, Minhas Ripudaman Singh (2021). A chair at the table: A scoping review of the participation of refugees in community-based participatory research in healthcare. Globalization and Health.

[CR17] Hajak VL, Sardana S, Verdeli H, Grimm S (2021). A systematic review of factors affecting mental health and well-being of asylum seekers and refugees in Germany. Frontiers in Psychiatry.

[CR18] Hashimoto-Govindasamy LS, Rose V (2011). An ethnographic process evaluation of a community support program with Sudanese refugee women in western Sydney. Health Promotion Journal of Australia.

[CR19] Hess JM, Isakson B, Githinji A, Roche N, Vadnais K, Parker DP, Goodkind JR (2014). Reducing mental health disparities through transformative learning: A social change model with refugees and students. Psychological Services.

[CR43] Kishi, K. (2017). *Assaults against Muslims in US surprass 2001 levels*. Pew Research Center. https://www.pewresearch.org/fact-tank/2017/11/15/assaults-against-muslims-in-u-s-surpass-2001-level/

[CR20] Moher D., Liberati A., Tetzlaff J., Altman D. G, PRISMA Group (2009). Preferred reporting items for systematic reviews and meta-analyses: The PRISMA statement. BMJ.

[CR21] Murray KE, Davidson GR, Schweitzer RD (2010). Review of refugee mental health interventions following resettlement: Best practices and recommendations. The American Journal of Orthopsychiatry.

[CR22] Onwuegbuzie AJ, Collins KMT, Frels RK (2013). Foreword: Using Bronfenbrenner’s ecological systems theory to frame quantitative, qualitative, and mixed research. International Journal of Multiple Research Approaches.

[CR23] Phillips D, Lauterbach D (2017). American muslim immigrant mental health: The role of racism and mental health stigma. Journal of Muslim Mental Health.

[CR24] Polcher K, Calloway S (2016). Addressing the need for mental health screening of newly resettled refugees: A pilot project. Journal of Primary Care & Community Health.

[CR25] Pottie K, Rahman HC, Ingleby P, Akl D, Russell EA, Ling G, Brindis D (2017). Building responsive health systems to help communities affected by migration: An international delphi consensus. International Journal of Environmental Research and Public Health.

[CR26] Riza E, Kalkman S, Coritsidis A, Koubardas S, Vassiliu S, Lazarou D, Linos A (2020). Community-Based Healthcare for Migrants and Refugees: A Scoping Literature Review of Best Practices. Healthcare.

[CR27] Salami B, Salma J, Hegadoren K (2019). Access and utilization of mental health services for immigrants and refugees: Perspectives of immigrant service providers. International Journal of Mental Health Nursing.

[CR28] Salt RJ, Costantino ME, Dotson EL, Paper BM (2017). "You are not alone” strategies for addressing mental health and health promotion with a refugee women’s Sewing group. Issues in Mental Health Nursing.

[CR29] Samari G, Alcalá HE, Sharif MZ (2018). Islamophobia, health, and public health: A systematic literature review. American Journal of Public Health.

[CR30] Samuels EA, White OL, Saadi EB, Padela A, Westerhaus A, Bhatt MD, Gonsalves G (2021). Health care utilization before and after the “Muslim Ban” executive order among people born in Muslim-majority countries and living in the US. JAMA Netwwork Open.

[CR31] Satinsky E, Fuhr DC, Woodward A, Sondorp E, Roberts B (2019). Mental health care utilisation and access among refugees and asylum seekers in Europe: A systematic review. Health Policy (Amsterdam Netherlands).

[CR32] Shah SFA, Hess JM, Goodkind JR (2019). Family separation and the impact of digital technology on the mental health of refugee families in the United States: Qualitative study. Journal of Medical Internet Research.

[CR33] Siddiq H, Rosenberg J (2021). Clinicians as advocates amid refugee resettlement agency closures. Journal of Public Health Policy.

[CR34] Slewa-Younan S, Guajardo MGU, Mohammad Y, Lim H, Martinez G, Saleh R, Sapucci M (2020). An evaluation of a mental health literacy course for Arabic speaking religious and community leaders in Australia: effects on posttraumatic stress disorder related knowledge, attitudes and help-seeking. International Journal of Mental Health Systems.

[CR35] Syed F, Keshavarzi S, Sholapur N, Keshavarzi H (2020). A survey of islamic clergy and community leaders regarding muslim mental health first responder training. Journal of Muslim Mental Health.

[CR36] Szaflarski M, Bauldry S (2019). The effects of perceived discrimination on immigrant and refugee physical and mental health. Advances in Medical Sociology.

[CR37] Uribe Guajardo MG, Slewa-Younan S, Kitchener BA, Mannan H, Mohammad Y, Jorm AF (2018). Improving the capacity of community-based workers in Australia to provide initial assistance to Iraqi refugees with mental health problems: An uncontrolled evaluation of a Mental Health Literacy Course. International Journal of Mental Health Systems.

[CR44] United Nations High Commissioner Refugees (2019). *Global trends: Forced displacement in 2018*. Retrieved from https://www.unhcr.org/5d08d7ee7.pdf

[CR38] Uribe Guajardo Maria Gabriela, Slewa-Younan Shameran, Kitchener Betty Ann, Mannan Haider, Mohammad Yaser, Jorm Anthony Francis (2018). Improving the capacity of community-based workers in Australia to provide initial assistance to Iraqi refugees with mental health problems: an uncontrolled evaluation of a Mental Health Literacy Course. International Journal of Mental Health Systems.

[CR39] van der Boor CF, White R (2020). Barriers to accessing and negotiating mental health services in asylum seeking and refugee populations: The application of the candidacy framework. Journal of Immigrant and Minority Health.

[CR40] Walker R, Koh L, Wollersheim D, Liamputtong P (2015). Social connectedness and mobile phone use among refugee women in Australia. Health & Social Care in the Community.

[CR41] Whittemore R, Knafl K (2005). The integrative review: Updated methodology. Journal of Advanced Nursing.

[CR42] Williams ME, Thompson SC (2011). The use of community-based interventions in reducing morbidity from the psychological impact of conflict-related trauma among refugee populations: A systematic review of the literature. Journal of Immigrant and Minority Health.

